# Evaluation of fasting plasma insulin and proxy measurements to assess insulin sensitivity in horses

**DOI:** 10.1186/s12917-021-02781-5

**Published:** 2021-02-15

**Authors:** Sanna Lindåse, Katarina Nostell, Peter Bergsten, Anders Forslund, Johan Bröjer

**Affiliations:** 1 Department of Clinical Sciences, Swedish University of Agricultural Sciences, Box 7054, 750 07 Uppsala, Sweden; 2 Department of Women’s and Children’s Health, Uppsala University, Uppsala, Sweden; 3 Department of Medical Cell Biology, Uppsala University, Uppsala, Sweden

**Keywords:** Endocrine, Equine metabolic syndrome, Beta‐cell response, Insulin dysregulation, Insulin resistance

## Abstract

**Background:**

Proxies are mathematical calculations based on fasting glucose and/or insulin concentrations developed to allow prediction of insulin sensitivity (IS) and β-cell response. These proxies have not been evaluated in horses with insulin dysregulation. The first objective of this study was to evaluate how fasting insulin (FI) and proxies for IS (1/Insulin, reciprocal of the square root of insulin (RISQI) and the quantitative insulin sensitivity check index (QUICKI)) and β-cell response (the modified insulin-to-glucose ratio (MIRG) and the homeostatic model assessment of β-cell function (HOMA-β)) were correlated to measures of IS (M index) using the euglycemic hyperinsulinemic clamp (EHC) in horses with insulin resistance (IR) and normal IS. A second objective was to evaluate the repeatability of FI and proxies in horses based on sampling on consecutive days. The last objective was to investigate the most appropriate cut-off value for the proxies and FI.

**Results:**

Thirty-four horses were categorized as IR and 26 as IS based on the M index. The proxies and FI had coefficients of variation (CVs) ≤ 25.3 % and very good reliability (intraclass correlation coefficients ≥ 0.89). All proxies and FI were good predictors of the M index (*r* = 0.76–0.85; *P* < 0.001). The proxies for IS had a positive linear relationship with the M index whereas proxies for β-cell response and FI had an inverse relationship with the M index. Cut-off values to distinguish horses with IR from horses with normal IS based on the M index were established for all proxies and FI using receiver operating characteristic curves, with sensitivity between 79 % and 91 % and specificity between 85 % and 96 %. The cut-off values to predict IR were < 0.32 (RISQI), < 0.33 (QUICKI) and > 9.5 µIU/mL for FI.

**Conclusions:**

All proxies and FI provided repeatable estimates of horses’ IS. However, there is no advantage of using proxies instead of FI to estimate IR in the horse. Due to the heteroscedasticity of the data, proxies and FI in general are more suitable for epidemiological studies and larger clinical studies than as a diagnostic tool for measurement of IR in individual horses.

## Background

Insulin dysregulation (ID) in horses is defined as any combination of fasting hyperinsulinemia, postprandial hyperinsulinemia or insulin resistance (IR) [1]. Endocrinopathic laminitis is associated with ID and it is therefore important to identify horses and ponies at-risk in order to prevent the development of clinical laminitis [2]. Several methods for diagnosing IR in horses have been established but the majority of them have been adapted from human medicine [3, 4]. Proxies are mathematical calculations based on fasting glucose concentration (FG) and/or fasting insulin (FI) concentrations developed to allow prediction of insulin sensitivity (IS) and β-cell response [4]. In both humans and horses, proxies correlate well with quantitative measurements of IS [5–7]. In a study by Treiber and coworkers [7] the reciprocal of the square root of insulin (RISQI), the quantitative insulin sensitivity check index (QUICKI), the modified insulin-to-glucose ratio (MIRG) and the homeostatic model assessment of β-cell function (HOMA-β) correlated well with either the IS or the acute insulin response to glucose (AIRg) quantified by the frequently sampled intravenous glucose tolerance test (FSIGTT). The comparisons were made in a group of 46 clinically healthy Thoroughbreds and Arabian horses. However, the use of proxies in horses has not been validated in horses with ID. In several species including the horse, IS and β-cell response are inversely related to each other [5, 8–10]. In humans, the proxies for IS are linearly correlated with quantitative measurements for IS, whereas proxies for β-cell response are inversely related [5, 6].

Whereas quantitative methods for assessing IS and β-cell response are expensive, time consuming and technically difficult to perform, proxies are inexpensive, easy to obtain and suitable for larger epidemiological studies and clinical trials [4]. Several proxies have been used in equine research [11, 12] but whether these proxies provide better information about the patients’ IS or β-cell response compared to the insulin and glucose concentrations they are calculated from has not yet been established. Insulin concentrations are influenced by feeding [13, 14] and therefore measurement of FI has been recommended to standardize testing [15]. However, use of FI to diagnose ID has been shown to have poor sensitivity [16]. On the other hand, a recent study concluded that FI had adequate sensitivity and specificity when using lower cut-off values than previously suggested [15].

The first objective of this study was to evaluate how FI and proxies for IS and β-cell response were correlated to quantitative measures of IS using the euglycemic hyperinsulinemic clamp (EHC) in a group of horses with insulin resistance (IR) and normal IS. A second objective was to evaluate the repeatability of FI and proxies in horses based on sampling on consecutive days. The last objective was to investigate the most appropriate cut-off value for the proxies and FI.

## Results

### Subject characteristics

**Fig. 1:**
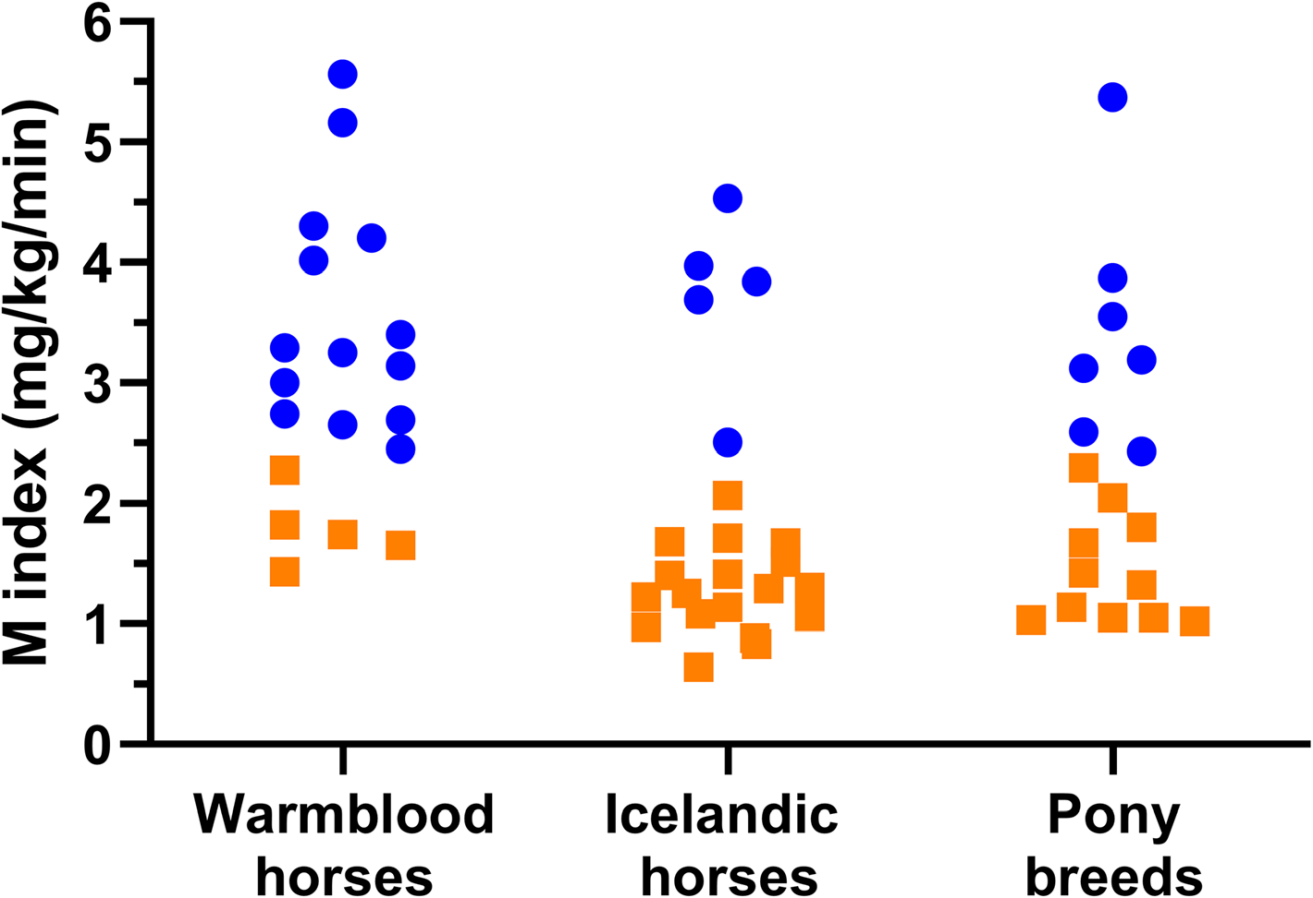
Scatterplots of individual horses’ insulin sensitivities (M index) determined by the euglycemic hyperinsulinemic clamp (*n *= 60) divided into 3 major groups of horses: Warmblood horses (*n* = 19); Icelandic horses (*n* = 23) and Pony breeds (*n* = 18). Horses with normal insulin sensitivity are marked with blue circles whereas horses with insulin resistance are marked with orange squares

Thirty-four horses were categorized as having IR (25 mares and 9 geldings) and 26 as having normal IS (15 mares and 11 geldings) based on the M index. The horses’ individual IS divided by group (warmblood horses, Icelandic horses and ponies) are shown in Fig. 1. Age (mean ± SD) was similar between the 3 groups of horses, *P* = 0.65 (13.2 ± 4.4, 12.5 ± 5.2 and 11.7 ± 4.8 years for the warmblood horses, Icelandic horses and pony breeds respectively). The groups of warmblood horses, Icelandic horses and pony breeds with IR had mean M indices of 1.8 ± 0.3, 1.3 ± 0.4 and 1.4 ± 0.5 respectively, with no differences between groups (*P* > 0.22). The corresponding means for the M indices of the IS horses within the same groups of horses were 3.6 ± 1.0, 3.7 ± 0.7 and 3.4 ± 1.0 respectively, with no differences between groups (*P* > 0.9).

### Correlation between proxies and insulin sensitivity measures from the EHC

**Fig. 2:**
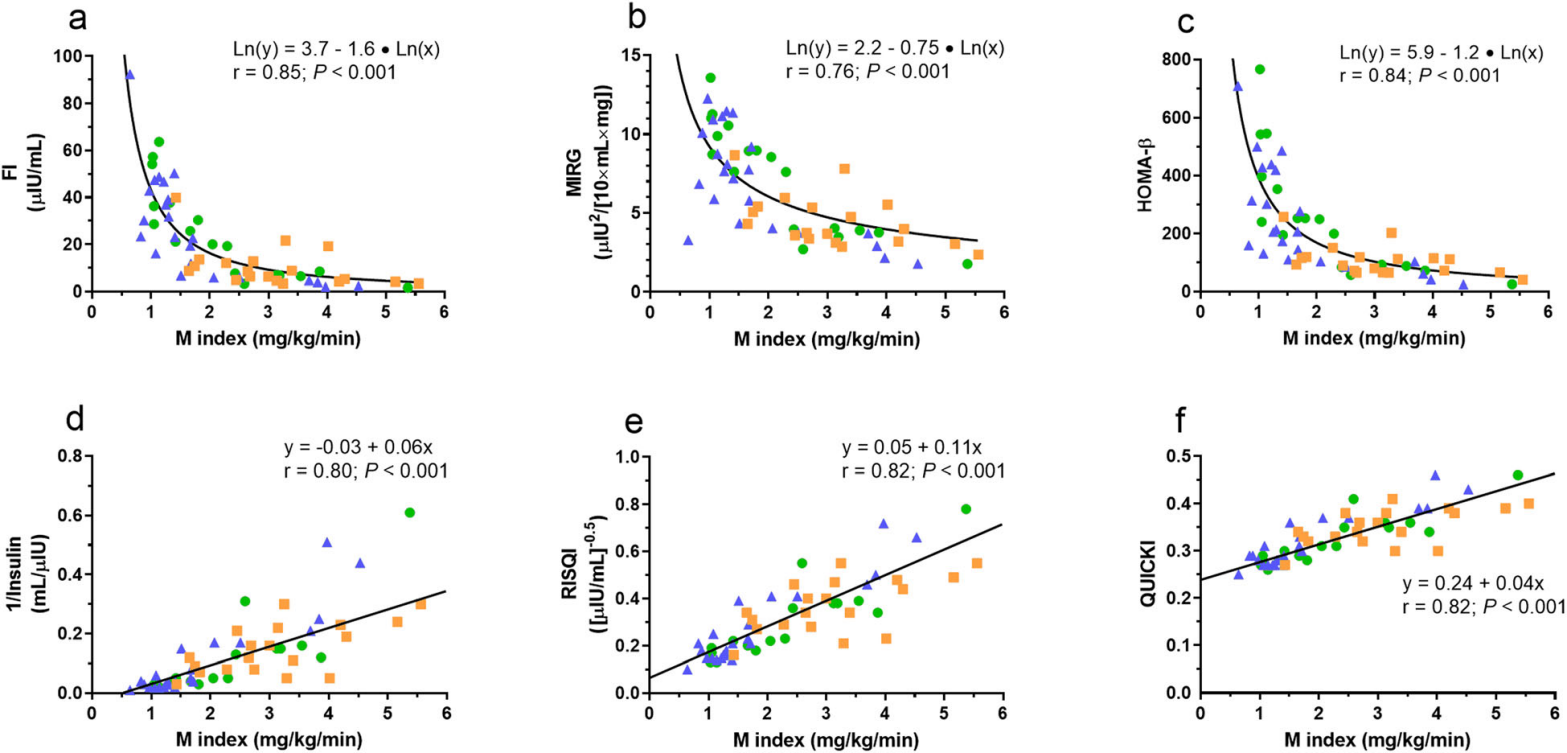
Scatterplots of individual horse data (*n* = 60) showing the relationship between fasting indices for β-cell response: FI, fasting insulin concentration (**a**); MIRG, modified insulin-to-glucose ratio (**b**) and HOMA-β, homeostatic model assessment of β-cell function (**c**) versus the whole body glucose disposal rate (M index) as well as the relationship between proxies for insulin sensitivity: 1/insulin (**d**); RISQI, reciprocal of the square root of insulin (**e**) and QUICKI, quantitative insulin sensitivity check index (**f**) versus M index. The line in **a** – **c** represents the regression line for log transformed data (Ln (y) = constant + β $\times$ Ln (x)), whereas the line in **d** – **f** represents the regression line for weighted linear regression. Orange squares represent warmblood horses, blue triangles represent Icelandic horses whereas green circles represent pony breeds

Scatter plots showing the correlation between fasting indices for β-cell response (FI, MIRG and HOMA-β) and the M index demonstrate an inverse relationship (Fig. 2, a - c). When IS decreased, the β-cell response expressed as the FI, MIRG or HOMA-β increased. The fasting indices for β-cell response were highly correlated with the M index derived from the EHC; *r* = 0.85, 0.76 and 0.84 for FI, MIRG and HOMA-β respectively (*P* < 0.001). A rectangular hyperbolic relationship was not evident for any relationship since β (95 % CI) did not include − 1 for any of the comparisons; -1.57 (-1.82 to -1.32), -0.75 (-0.92 to -0.59) and − 1.25 (-1.46 to -1.03) for FI, MIRG and HOMA-β respectively.

Scatter plots showed linear correlation between proxies for IS (1/insulin, RISQI and QUICKI) and the M index (Fig. 2, d – f). Visual inspection of scatterplots for 1/insulin and RISQI (Fig. 2, d and e) show heteroscedasticity. The proxies for IS were highly correlated with the M index derived from the EHC; *r* = 0.80, 0.82 and 0.82 for 1/insulin, RISQI and QUICKI respectively (*P* < 0.001).

### Bland‐altman plots, within‐subject variability and reliability

**Fig. 3:**
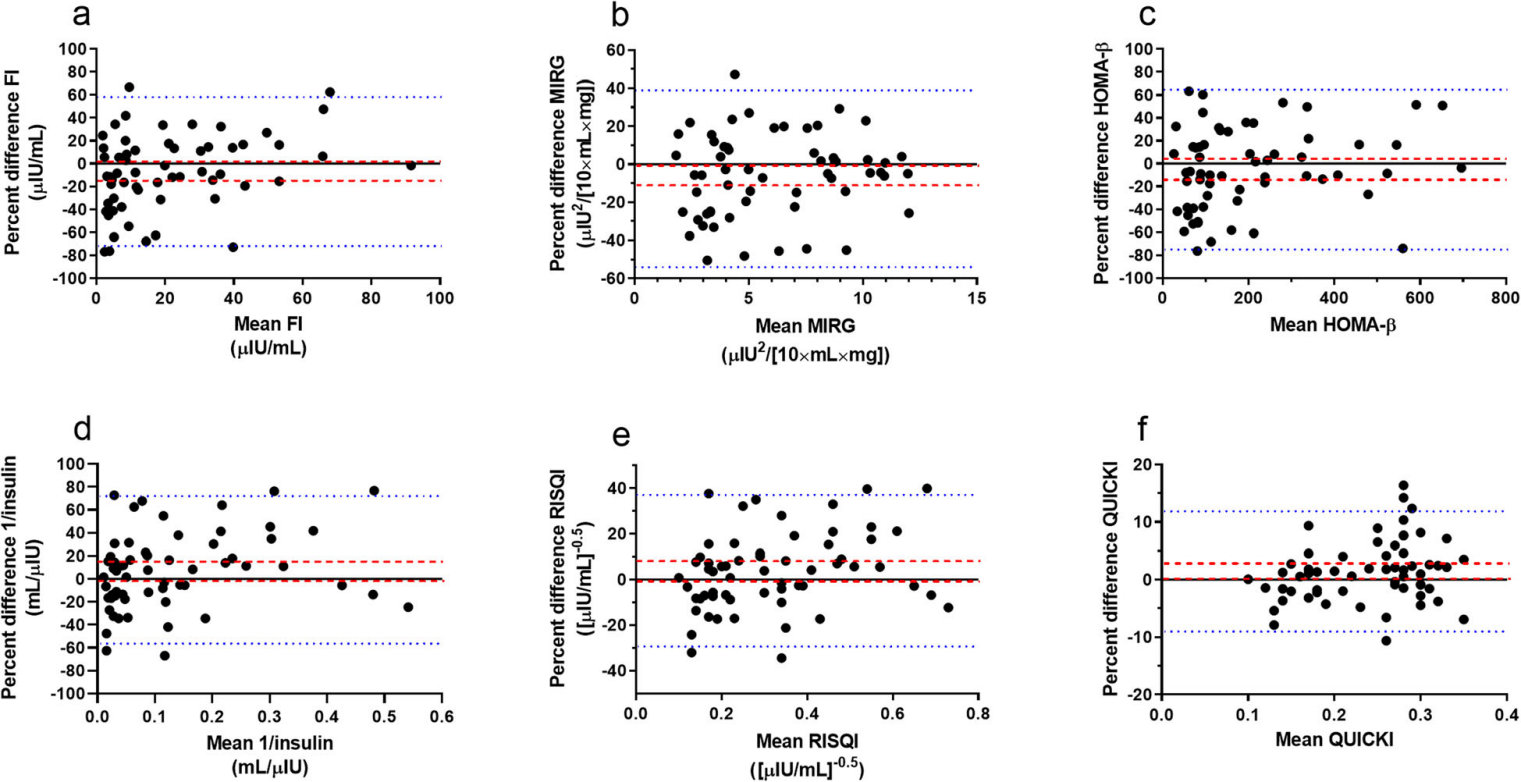
Relative difference Bland-Altman plots where test and retest values are divided by their means and expressed as percentage are plotted against their means for fasting indices for β-cell response: FI, fasting insulin concentration (**a**); MIRG, modified insulin-to-glucose ratio (**b**); HOMA-β, homeostatic model assessment of β-cell function (**c**) and for insulin sensitivity: 1/insulin (**d**); RISQI, reciprocal of the square root of insulin (**e**); QUICKI, quantitative insulin sensitivity check index (**f**). The blue dotted line represents limits of agreement and the dashed red line represents the bias for repeated measures

Bland-Altman plots for proxies and FI are presented in Fig. 3. All data are presented in relative difference Bland-Altman plots to correct for increasing measurement error proportional to the mean (heteroscedasticity). The 95 % CI for the mean of the difference between study days did include 0 for tested proxies except for MIRG, although the 95 % upper CI for MIRG was very close to 0 (-0.9), suggesting a relatively small effect (bias) of the day of study.

Within subject variability between days was calculated and the CVs for fasting indices are presented in Table 1. The proxies RISQI and QUICKI showed low within-subject variability (CV of ≤ 12.2 %). The fasting indices for β-cell response (FI, MIRG, HOMA-β) demonstrated higher within-subject variability (CV between 17.5 and 25.3 %). The ICC (reliability) for fasting indices are presented in Table 1. All proxies and FI showed excellent ICCs (≥ 0.89). There was a significant difference in MIRG between day 1 and day 2 (*P* = 0.03).

**Table 1:** Repeatability and reliability for fasting indices obtained from insulin and glucose concentrations measured on two consecutive days in 60 horses

Variable	Day 1 Median (IQR)	Day 2 Median (IQR)	CV% (CI)	ICC	CR%	Paired t-test (between-test days) *P* value
**Insulin sensitivity**:						
1/insulin (mL/µIU)	0.088 (0.032–0.22)	0.076 (0.032–0.17)	23.8 (19.2–27.7)	0.95	65	0.10
RISQI ([µIU/mL]^−0.5^)	0.30 (0.18–0.47)	0.28 (0.18–0.41)	12.2 (9.7–14.2)	0.95	33	0.14
QUICKI	0.33 (0.28–0.39)	0.32 (0.28–0.37)	3.9 (2.9–4.6)	0.94	10	0.06
**β-cell response**:						
FI (µIU/mL)	11.4 (4.7–31.9)	13.2 (6.0–31.5)	23.8 (19.2–27.7)	0.95	65	0.11
MIRG ([µIU]^2^ /[10$\times$mL$\times$mg)	4.9 (3.1–8.5)	13.2 (6.0–8.8)	17.5 (13.0–21.1)	0.89	47	0.03
HOMA-β	126 (75–306)	125 (81–257)	25.3 (21.3–28.7)	0.91	70	0.26

*RISQI* reciprocal of the square root of insulin; *QUICKI* quantitative insulin sensitivity check index; *FI* fasting insulin concentration; *MIRG* modified insulin-to-glucose ration; *HOMA-β* homeostatic model assessment of β-cell function; *IQR* interquartile range; *CV* coefficient of variation; *ICC* intraclass correlation coefficient; *CR* coefficient of repeatability

### Cut‐off values for proxies to predict insulin resistance

**Fig. 4:**
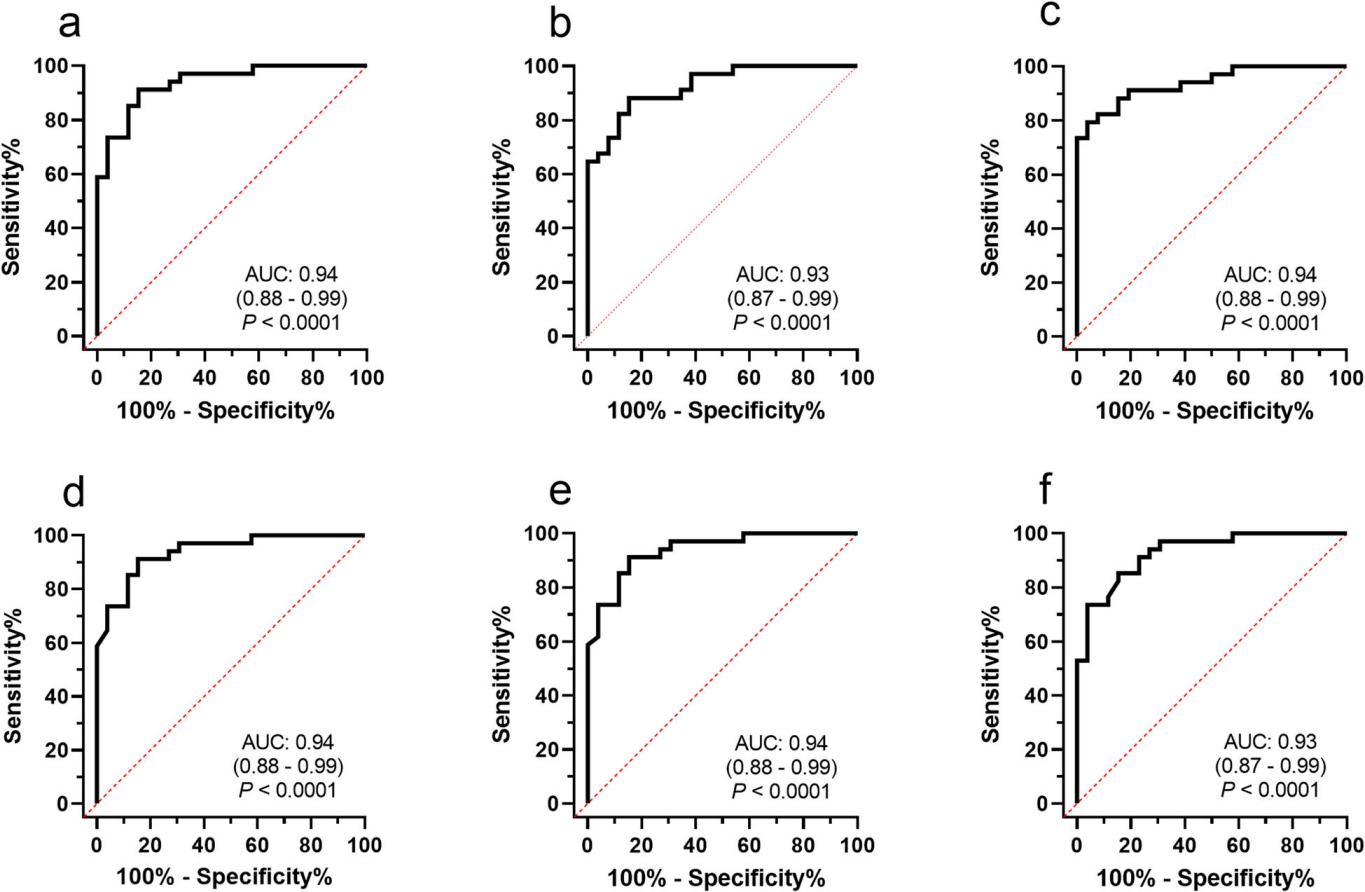
ROC curves for fasting indices for β-cell response: FI, fasting insulin concentration (**a**); MIRG, modified insulin-to-glucose ratio (**b**); HOMA-β, homeostatic model assessment of β-cell function (**c**) and for insulin sensitivity: 1/insulin (**d**); RISQI, reciprocal of the square root of insulin (**e**); QUICKI, quantitative insulin sensitivity check index (**f**) using the M index from the euglycemic hyperinsulinemic clamp as the reference. The red dotted diagonal line represents a completely uninformative test, wherein the area under the curve (AUC) is 50 %. The area under the ROC curve with confidence interval (CI), represented by the thicker line, is reported in each graph. The P value is reported for the test of the null hypothesis that the AUC equals 0.50

ROC curves for proxies and FI are presented in Fig. 4. The ROC curve analysis resulted in an optimal cut-off value for FI of > 9.5 µIU/mL to predict IR based on the Mercodia Equine insulin ELISA. This cut-off value was based on the Youden’s index for maximizing sensitivity and specificity in the investigated population, with sensitivity and specificity of 91 % and 85 % respectively. The cut-off values to distinguish horses with IR from horses with IS using the proxies MIRG, HOMA-β, 1/insulin, RISQI and QUICKI are reported in Table 2. Due to the non-linear inverse relationship between β-cell response and IS, values higher than the cut-offs indicate IR for indices estimating the β-cell response (FI, MIRG, HOMA-β) whereas lower values than the cut-offs indicate IR for proxies estimating IS (1/insulin, RISQI, QUICKI). The sensitivity for these fasting indices ranged between 79 % and 91 %, whereas the specificity ranged between 85 % and 96 % in the studied population (Table 2).

**Table 2:** Calculated cut-off values for fasting indices based on receiver operating characteristic curve and Youden’s index analyses using the euglycemic hyperinsulinemic clamp (M index) as the reference for diagnosing insulin resistance

Variable	Cut-off values to predict IR	AUC	Sn% (95 % CI)	Sp% (95 % CI
**Insulin sensitivity**:				
1/Insulin (mL/µIU)	< 0.11	0.94 (0.88–0.99)	91 (77–97)	85 (66–94)
RISQI ([µIU/ml]^−0.5^)	< 0.32	0.94 (0.88–0.99)	91 (77–97)	85 (66–94)
QUICKI	< 0.33	0.93 (0.87–0.99)	85 (70–94)	85 (67–94)
**β-cell response**:				
FI (µIU/mL)	> 9.5	0.94 (0.88–0.99)	91 (77–97)	85 (66–94)
MIRG ([µIU_ins_]^2^ /[10•L• mg_gluc_])	> 4.4	0.93 (0.87–0.99)	88 (73–95)	85 (66–93)
HOMA-β	> 147	0.94 (0.88–0.99)	79 (63–90)	96 (81–100)

*RISQI* reciprocal of the square root of insulin; *QUICKI* quantitative insulin sensitivity check index; *FI* fasting insulin concentration; *MIRG* modified insulin-to-glucose ration; *HOMA-β* homeostatic model assessment of β-cell function; *AUC* area under the curve; *Sn* sensitivity; *Sp* specificity; *CI* confidence interval

## Discussion

The present study demonstrated that all investigated proxies and FI were strongly correlated with quantitative measures of the horses’ IS using the M index of the EHC. However, these correlations had 2 major types of structure; curvilinear and linear. There was a curvilinear inverse relationship between fasting indices for the β-cell response (FI, MIRG or HOMA-β) and the M index whereas the relationship was linear between proxies for IS (1/insulin, RISQI or QUICKI) and the M index. We observed that proxies and FI in general demonstrated reasonably good repeatability and very high reliability. Cut-off values to predict IR were established for all proxies and FI using ROC curve analyses to maximize sensitivity and specificity. The sensitivity for these fasting indices in the investigated population ranged between 79 and 91 % and the specificity ranged between 85 and 96 %.

Kahn and coworkers [5] have reported a non-linear inverse relationship between FI and IS in humans. A hyperbolic relationship has been reported between the β-cell response and IS in several species including the horse [5, 8, 17, 18]. A curvilinear inverse relationship was also found between FI and IS determined by the EHC in this study, but it was not found to be hyperbolic. Fasting insulin concentrations are thus an estimate of β-cell response for the steady state period during the post absorptive phase but the estimate is different than those obtained from studies in which secretion is stimulated by exogenous glucose e.g. oral glucose tolerance tests, hyperglycemic clamp and FSIGTT [17, 19]. During basal (fasting) steady-state condition, glucose concentration is dependent on insulin-regulated hepatic glucose production and glucose removal by tissues, whereas insulin concentration is dependent on the β-cell response to glucose originating from the liver and insulin removal [4]. Increased FI therefore reflect both hepatic IR, increased insulin secretion as well as metabolic clearance of insulin. Notably, FI do not take into account the effect of decreased functional β-cell mass. Fasting insulin concentrations thus give erroneous estimates for β-cell function in patients with diabetes mellitus type 2 [4]. In the horse with ID, hyperinsulinemia is the typical manifestation [1] and not diabetes mellitus type 2, suggesting that FI can be used for diagnosing ID without a correction factor using FG. In humans, the HOMA model is used to estimate β-cell function from FI and FG. The feedback loop between endogenous glucose production in the liver and the β-cell response of insulin secretion is central to the model. The HOMA-β evaluates the β-cell function by calculating the ratio of FI to FG after numeric adjustment of the values with specific constants [20]. In a previous study, HOMA-β as well as MIRG were found to correlate well to the AIRg during an FSIGTT, a measure of the early insulin response to intravenous glucose [7]. In fact, these two proxies for β-cell response gave similar results in terms of specificity, sensitivity and total predictive power. However, one problem with MIRG is that horses with hypersinulinemia > 50 µIU/mL have erroneously low MIRG values. This is likely the reason for the lower non-linear correlation to IS for MIRG compared to FI or HOMA-β in the present study. When values > 50 µIU/mL were excluded (*n* = 5) from the analysis the correlation coefficient (r) increased from 0.76 to 0.82. Thus, the formula for MIRG is only valid for horses with FI ≤ 50 µIU/mL, which limits its use in horse populations with ID. An alternative to MIRG has been suggested by using an adapted MIRG for ponies (modified insulin-to-glucose ratio for ponies; MIGRP) [11]. When the adjusted MIRG proxy was adapted to all our data, MIGRP correlated well to the M index (Ln(y) = 1,7–1.3 $\times$Ln(x); *r* = 0.82, *P* < 0.001; scatterplot data not shown) and yielded a scatterplot pattern very similar to that of FI versus M index or HOMA-β versus M index. The fasting indices for β-cell response in this study, FI, MIRG and HOMA-β, had a curvilinear inverse relationship with IS and they can therefore be used not only to estimate the β-cell response but also the IS. The simplest index FI had the best non-linear inverse correlation with the M index. In this study, we did not quantify the β-cell function, and we can therefore not draw specific conclusions on how well FI, MIRG and HOMA-β are able to estimate the β-cell response in the horse. This is a limitation with the study since it would have been valuable not only to compare fasting indices with quantitative measures of IS but also with different aspects of the β-cell response using, for example, the hyperglycemic clamp [19]. However, this would have complicated the study and it was not feasible since we used clinical cases in our study.

Due to the non-linear inverse relationship between FI and IS in patients with preserved β-cell function, mathematical transformation of FI into 1/insulin provides an estimate of IS. This proxy demonstrated a very good positive linear correlation with the M index from the EHC in the present study but data showed heteroscedasticity. Further mathematical transformation by taking the square root of the denominator (RISQI) improved the heteroscedasticity to a certain extent but it had a marginal effect in improving the correlation. In humans, QUICKI was developed by Katz and coworkers [6] to obtain a proxy that could be used in both diabetic and non-diabetic subjects. By log transformation to both FI and FG in the denominator a correction for patients with diabetes mellitus type 2, where glucose is high and insulin is low, was established. As FI has a skewed distribution, log transformation of insulin in the mathematical formula of QUICKI improved the linear correlation with the M index of the EHC with decreased heteroscedasticity in the present study compared to the proxy 1/insulin. Similar results with minimal heteroscedasticity and very good correlation with the M index have been found for man [6]. If the QUICKI proxy is applicable to equine patients with diabetes mellitus type 2 has, however, not been evaluated.

The fasting indices for β-cell response had CV values between 17.5 % and 25.3 % and ICCs ≥ 0.89. These results are comparable with the results of repeatability for peak insulin concentration and AUC for insulin from the oral sugar test in a previous study (mean CV of 18.3 % and 16.9 % respectively and ICC of 0.83 and 0.91 respectively) [21]. However, the data for CV are not directly comparable since the aforementioned study used mean CVs with a bias with lower values [22]. In comparison, dynamic tests that more specifically measure the β-cell response such as the FSIGTT (AIRg) [23] and the hyperglycemic clamp (AUC for insulin) [19] demonstrate much lower CVs and somewhat higher ICC (≤ 11.7 % with ICC ≥ 0.93). In contrast, proxies for IS demonstrated lower CVs than the fasting indices for β-cell response. In comparison with a previous study [24] the indices FI, RISQI and MIRG in the present study had better CVs. One explanation is that sampling in this study was performed early in the morning just before feeding, which gave standardized basal (fasting) conditions. In the aforementioned study, sampling was done at 12 AM and hay was available for horses before sampling [24], which may have affected the variability in test results. The CR is an index that quantifies the repeatability [25]. The relative differences between test and retest values and their means were used in order to obtain normal distribution, and the CR was therefore expressed in percentage. Since the percentage error was distributed normally, a 95 % confidence interval could be calculated around a measurement. The CR is thus the smallest difference in percentage between two measurements that can be interpreted as a real difference and not measurement error [25]. Only QUICKI demonstrated a low CR (10 %). The other fasting indices had much higher relative CR (30–70 %).

Previous studies have demonstrated poor sensitivity of FI to diagnose ID [16, 26]. However, the sensitivity and specificity of a test in a population is affected by the cut-off value used. In general, reducing the cut-off value improves the sensitivity but decreases the specificity. In a recent study, the authors demonstrated that the poor sensitivity for FI was related to the cut-off value used rather than FI being an intrinsically inappropriate test [15]. By reducing the commonly suggested cut-off value of 20 µIU/mL to 5.2 µIU/mL for FI, the sensitivity for the diagnosis of ID increased from 15 to 63 % without a major impact on the specificity, which decreased from 100 to 87 % [15]. In comparison, the cut-off value for FI in the present study was 9.5 µIU/mL with sensitivity of 91 % and specificity of 85 %. The higher cut-off value in the present study can be explained by the use of different techniques for analyses of insulin in the two studies. The chemiluminescent assay has found to give lower readings for endogenous equine insulin compared to the species optimized equine ELISA used in the present study [27]. The higher sensitivity for FI in the present study may reflect the study population with an evenly distributed wide range of IS with relatively fewer sampling points around the cut-off level for FI compared with the study by Olley and coworkers [15]. Compared to FI, the proxies gave similar results for predicting IR with sensitivities of 79 to 91 % and specificities varying between 85 to 96 %. It has been suggested that blood samples for insulin concentrations should be obtained after feeding and not in the morning after feed withdrawal overnight in order to increase the sensitivity of the test [28]. However, the postprandial insulin response after feeding different types of forage vary in magnitude and duration leading to variable insulin results [13, 14]. The horses in this study were sampled for glucose and insulin in the morning at 7 AM before feeding to establish stable basal conditions. This is not always possible in clinical practice and further research is needed to determine the ideal period of feed withdrawal after the morning feed for sampling of insulin and glucose later during the day.

## Conclusions

Proxies are mathematical calculations based on FI or the combination of FI and FG with the following principles for adjustment in horses with preserved β-cell function. When FI appears in the numerator of the calculation the proxy predicts the β-cell response whereas, if FI appears in the denominator, the proxy predicts IS. This is based on the curvilinear inverse relationship between insulin sensitivity and the β-cell response. Thus, proxies will in fact be able to predict IS regardless of whether they are intended to estimate the β-cell response or the IS. We have shown that in a population of horses with a broad range of IS, no proxy was superior to the others or to FI. However, the QUICKI showed an additional advantage compared to the other proxies by offering lower CVs and CRs. In addition, MIRG is not recommended for use in ID horses due to limitations with the mathematical formulae. Thus, there is no advantage in using proxies instead of FI to estimate IR in horses with preserved β-cell function. Due to the heteroscedasticity of the data, proxies and FI in general are more suitable for epidemiological studies and larger clinical studies than as a diagnostic tool for measurement of IR in individual horses. Finally, it is important to point out that the cut-off values for FI and proxies presented in this paper are only valid for analyses based on the Mercodia’s species optimized equine ELISA since different insulin assays give different results for equine insulin.

## Methods

### Horses

All horse owners provided informed written consent. Thirty-five client owned horses and ponies previously diagnosed with ID by referring veterinarians using an oral sugar test [21] were enrolled in the study. The diagnosis of ID was based on blood samples obtained between 60 and 90 minutes after oral administration of the syrup and analyzed for insulin (Mercodia equine insulin ELISA, Mercodia AB, Uppsala, Sweden). To be eligible to participate in the study the insulin concentration had to be > 90 µIU/mL (insulin concentrations > 45 µIU/mL is considered diagnostic for ID). In addition, 26 clinically healthy horses and ponies owned by the Swedish University of Agricultural Sciences were included in the study. The horses were divided into 3 major groups: warmblood horses (warmbloods and Standardbreds), Icelandic horses and ponies (pony crossbreds, Welsh ponies, Gotland ponies and Shetland ponies). Criteria for inclusion were no ongoing episode of laminitis based on clinical examination and normal plasma ACTH adjusted for the season. All horses in the study were fed a hay or haylage diet supplemented with minerals. Horses were housed in individual box stalls and allowed daily turnout in a dirt or sand paddock. None of the horses had been on grass pasture for at least 2 months before testing.

### Experimental design

All horses were acclimatized for at least 48 hours to the environment where sampling was to take place. After acclimatization, horses were sampled for FI and FG at 7 AM on two consecutive days immediately followed by an EHC the second day. Blood sampling for FI and FG as well as the EHC took place after feed withdrawal overnight.

### Proxy measurements

The day before testing an IV catheter (Intranule, 2.0 × 105 mm. Vygon, Ecouen, France) for blood sampling was inserted into one of the jugular veins under local anesthesia (EMLA, AstraZeneca AB, Södertälje, Sweden). Blood samples for FI and FG were collected from the jugular catheter at 7 AM on two consecutive days. Fasting insulin and glucose concentrations were used to calculate proxies. Insulin sensitivity was estimated using the proxies 1/Insulin [4], RISQI [7] and QUICKI [6] whereas the β-cell response was estimated using FI and the proxies MIRG [7] and HOMA-β [20].

Proxies were calculated using the following formulae:

$$\mathrm{RISQI}\;=\;{\lbrack\mathrm{Insulin}\rbrack}^{-0.5}\;=\;(1/\sqrt{\left[\text{Insulin}\right]})$$

$$\mathrm{QUICKI}\:=\:1/(\log\lbrack\mathrm{Insulin}\rbrack\:+\:\log\lbrack\mathrm{Glucose}\rbrack)$$

$$\mathrm{MIRG}\;=\;(800\;‒\;0.3\;\times\;{(\lbrack\mathrm{Insulin}\rbrack\;‒\;50)}^2)/(\lbrack\mathrm{Glucose}\rbrack\;‒\;30)$$

$$\mathrm{HOMA}-\mathrm\beta\;=\;(20\;\times\;\lbrack\mathrm{Insulin}\rbrack)/(\lbrack\mathrm{Glucose}\rbrack\;--\;3.5)$$

Glucose concentrations expressed in the SI-unit mmol/L were used for HOMA-β but were converted into mg/dl before insertion into the formulae for QUICKI and MIRG. Insulin concentration in µIU/mL were used in all calculations.

### EHC – euglycemic hyperinsulinemic clamp

A second IV catheter (Intranule, 2.0 × 105 mm. Vygon, Ecouen, France) for infusions was inserted under local anesthesia (EMLA, AstraZeneca AB, Södertälje, Sweden) into the contralateral jugular vein in the afternoon on the day preceding the EHC. Blood samples for determination of FI and FG were drawn from the sampling IV catheter immediately before the start of the EHC at 7 AM. The EHC procedure has previously been described for use in horses [23, 29]. A continuous rate infusion of regular insulin (Humulin Regular, Eli Lilly Sweden AB, Solna, Sweden) was maintained throughout the 180 min clamp procedure at 3 mIU/kg/min. Blood glucose was kept at 5 mmol/L using a variable continuous rate infusion of glucose (Glucose Fresenius Kabi 500 mg/ml, Fresenius Kabi AB, Uppsala, Sweden). Adjustment of the glucose infusion was made based on the results of measurement (Accu-Check Aviva, Roche Diagnostics Scandinavia AB, Bromma, Sweden) of blood glucose concentration performed every 5 minutes. Serial blood samples were obtained every 10 minutes during the clamp for later analyses of plasma glucose to enable calculation of whole body glucose uptake, i.e. metabolic rate of glucose (M index). The steady-state period of the clamp was defined as the last 60 minutes. The M index was defined as the infusion rate of exogenous glucose administered during the steady state after correction of the glucose space [23, 29]. Horses were classified as IR if their M index was < 2.4 mg/kg/min. This cut-off level was based on the normal distribution of the M index (mean and 95 % confidence interval; 3.8 (2.4–5.2) in a group of metabolically healthy control horses (Icelandic horses and Gotland ponies) from a previous study [30]. The lower confidence interval for the M index in this group of horses was used as the cut-off for IR in the present study.

### Analysis of blood samples

All blood samples were collected into evacuated tubes (Vacuette 9 ml, Greiner Bio-One GmbH, Kremsmünster, Austria) containing lithium heparin and immediately placed on ice for 5 minutes before centrifugation (10 min, 2700 × g). Plasma was separated, frozen rapidly and stored at -80°C until later analysis of plasma insulin and glucose concentrations. Plasma glucose concentrations were measured enzymatically with an automated clinical chemistry analyser (YSI 2300 Stat Plus Analyzer, YSI Incorporated, Yellow Spring, Ohio). Endogenous concentration of plasma insulin was measured using a commercialised equine-optimised ELISA (Mercodia equine insulin ELISA, Mercodia AB, Uppsala, Sweden) and insulin concentrations were verified with a commercial kit (Mercodia animal insulin control; low, medium and high, Mercodia AB, Uppsala, Sweden) [31]. All analyses were performed in duplicate.

### Statistical analysis

All data were analyzed using a commercially available software program (JMP® Pro, version 15.0.0, SAS Institute Inc, Cary, North Carolina). The age of the horses and their M index were compared between groups of horses using one-way ANOVA. Comparisons between groups were performed by use of the Tukey-Kramer post hoc test. Variables were tested for homogeneity of variance using the Levene’s test. All residuals were analyzed for normality using the Shapiro-Wilk test. Data are presented as mean ± standard deviation (SD).

According to Bergman’s hypothesis insulin secretion is inversely related to IS in a rectangular hyperbolic relationship; y = constant $\times$ 1/x, where y and x represent β-cell response and IS respectively [5, 9]. This equation can be re-expressed through a log-transformation to a linear model: Ln (y) = constant + β $\times$ Ln (x), where β is the regression coefficient. If β was close to -1 (if the 95 % CI for β included − 1 but excluded 0) the rectangular hyperbolic relationship was considered to be fulfilled. This linear function was used to describe the relationship between fasting indices for β-cell response (FI, MIRG and HOMA-β) and IS (M index).

The proxies for IS (1/Insulin, RISQI and QUICKI) were initially compared with quantitative measurement of IS (M index) as an independent variable using simple linear regression. The residuals were normally distributed but showed heteroscedasticity and the regression model was therefore changed to weighted linear regression.

Bland-Altman plots of absolute differences between test and retest values against their mean were initially used to assess for systematic bias and uniform data distribution. The absolute difference plots demonstrated heteroscedasticity and data were therefore presented in relative difference Bland-Altman plots where the difference between test and retest values were divided by their means, expressed as percentage, and then plotted against their mean [32]. The relative differences between test and retest values were assessed for normal distribution using the Shapiro-Wilk test. The 95 % CI for the mean relative differences between test and retest values was calculated. If the 95 % CI included 0, no systematic bias was evident. Coefficient of repeatability (CR) was calculated from the SD of the relative differences (absolute value of 1.96 $\times$ SD) and expressed as percentage [25]. The CVs were calculated using the root mean square method and reported with 95 % confidence interval [22]. The intra-class correlation coefficient (ICC) was computed for all fasting indices using a one-way random effects model ANOVA on log transformed data to ensure normal distribution among residual. Comparison between fasting indices sampled at day 1 and day 2 were performed using a paired t-test on log transformed data to ensure normal distribution among all data. Values of *P* < 0.05 were considered as significant for all analyses.

Receiver operating characteristic (ROC) curve and Youden’s index analysis were used to determine the optimal cut-off for all proxies and FI with M index as the reference. Sensitivity, specificity and area under the curve (AUC) were calculated for each ROC curve analysis and reported with a 95 % CI.

## Data Availability

The datasets analysed during the current study are available from the corresponding author on reasonable request.

## Abbreviations

AIRg: Acute insulin response to glucose
AUC: Area under the curve
CV: Coefficient of variation
EHC: Euglycemic hyperinsulinemic clamp
EMS: Equine metabolic syndrome
FG: Fasting glucose
FI: Fasting plasma insulin concentrations
FSIGTT: Frequently sampled intravenous glucose tolerance test
HOMA-β: Homeostatic model assessment of β-cell function
ID: Insulin dysregulation
IR: Insulin resistance
IS: Insulin sensitivity
M index: Whole body glucose disposal rate
MIRG: Modified insulin-to-glucose ratio
QUICKI: Quantitative insulin sensitivity check index
RISQI: Reciprocal of the square root of insulin
SD: Standard deviation
